# Agronomic and economic performance of mung bean (*Vigna radiata* L.) varieties in response to rates of blended NPS fertilizer in Kindo Koysha district, Southern Ethiopia

**DOI:** 10.1515/biol-2022-0461

**Published:** 2022-09-08

**Authors:** Mulu Baza, Dereje Shanka, Mesfin Bibiso

**Affiliations:** Wolaita Zone Agricultural Department, Wolaita Zone Agricultural Office, Wolaita Sodo, Ethiopia; Department of Plant Science, Wolaita Sodo University, P.O. Box 238, Wolaita Sodo, Ethiopia; Department of Chemistry, Wolaita Sodo University, P.O. Box 238, Wolaita Sodo, Ethiopia

**Keywords:** productivity, low-fertility, yield, yield attribute, net return

## Abstract

Mung bean is one of Ethiopia’s most important pulse crops in the lowlands. The main constraints to mung bean productivity in Ethiopia are low soil fertility and improved varieties. During the 2018 cropping season, a field experiment was conducted in Kindo Koysha woreda with the objective of evaluating the effects of NPS fertilizer rates on yield and yield attributing traits of four mung bean varieties. Treatments consisted of factorial combinations of four mung bean varieties (N26, Shewarobit, NVL-1, and Chinese) with four NPS fertilizer rates (0, 50, 100, and 150 kg ha^−1^) laid out in randomized complete block design with three replications. The combination of the N26 variety with 150 kg NPS produced the highest number of pods per plant (15.46), seeds per pod (10.93), grain yield (1240.70 kg ha^−1^), and biomass (3177.40 kg ha^−1^). Moreover, the combination of 100 kg NPS ha^−1^ with the variety N26 also generated the highest net return (31,734.30 Birr ha^−1^) with a marginal rate of return of 771.71%. Thus, it may be tentatively stated that the usage of 100 kg NPS ha^−1^ with the variety N26 was determined to be optimum for the development of mung bean in the study region.

## 1 Introduction

Mung bean (*Vigna radiata* L.) is a Fabaceae plant that is one of the most important legume crops. It grows in tropical and subtropical regions around the world [1]. Mung bean is widely cultivated for human food consumption; it can be used as green manure and livestock feed. On average, mung bean seeds contain 26% protein, 62.5% carbohydrates, 1.4% fiber, vitamins, minerals, calcium, and phosphorus. Because they are easy to digest, they replace the scarce animal protein in the human diet in tropical regions of the world [2]. The most common characteristics of this crop are its short life cycle and ability to carry out biological nitrogen fixation that satisfies the nitrogen demand of the crop [3]. Due to its rapid growth and early maturity, this crop can be used to improve planting patterns because it can be planted as a catch and intercrop crop. It can be mainly planted in crop rotation with cereals [4].

Despite its multiple uses, mung bean is considered a newly introduced crop in Ethiopia. It is grown locally as “Masho” and is grown in few areas of the country; the production is also very small, mainly in the North Shewa and South Wollo Zones of the Amhara region, Southern Nations, Nationalities, and People’s Region (SNNPR) (Gofa, Konso, and Derashe), Tigray Regions (Humara), Oromia Regions (Harrag), and some woredas in Benshangul Gummuz Region [5]. According to the Central Statistical Agency report [5], the area covered by Ethiopian mung bean in 2017/18 was 41630.20 ha, the average grain yield was 1,235 kg ha^–1^, and the annual output was 51,413,297 kg. The area coverage of the crop in the SNNPRS is estimated to be 224.4 ha, and the average productivity is 931 kg ha^–1^, which is far below the potential yield level of 1,500 kg ha^–1^ [6].

The reasons for the low productivity of mung beans in Ethiopia include biological and non-biological factors as well as other production restrictions. Among them, fertilizer management is an important factor affecting the growth and yield of mung beans [7]. However, productivity in the area is still low due to a number of production constraints, including lowland agronomic management practices, adequate nutrient supply, disease, poor ventilation, unbalanced nutrient supply, and a lack of high-yielding varieties. Among them, nutrient deficiency is the biggest factor limiting the yield of beans in Ethiopia. Nitrogen (N), phosphorus (P), and trace elements such as sulfur (S) are important factors that have a greater impact on the growth, development, and yield of mung beans [7]. In line with this, low production of mung bean has been shown in growing areas of the country as a result of limited nutrient supply [8]. In order to address these problems, maintaining soil fertility through a balanced fertilization program, including chemical, organic, and biological fertilizers, is quite important in the yield and quality improvement of agricultural crops [9,10,11].

Nitrogen is an important mineral whose nutritional management requires special attention due to its diverse roles in plant physiology and metabolites biosynthesis, as well as its dynamics in soil [12,13,14,15]. Mung beans require more N in the reproductive stage than in the vegetative stage. Moreover, P is the second most important phytonutrient in crop production after N. Phosphorus deficiency is exacerbated under dryland conditions, which affect fertilizer efficiency and successful crop production [16]. Sulfur promotes the formation of legume nodules and stimulates the production of seeds. With the application of S up to 20 kg ha^−1^, the total number of nodules and active nodules increased significantly [17]. The soil fertility map of the study area showed that levels of N, P, K, S, and Zn, as well as elements such as B and Cu, are depleted, and deficiency symptoms are observed in major crops [18].

Research done so far to solve the problems in the study area and elsewhere in the country on the response of legumes, including mung beans, to blended NPS fertilizer showed promising results [19,20]. However, farmers in the study area have been using 100 kg of DAP ha^−1^ (18 kg N and 46 kg P_2_O_5_ ha^−1^) for all legumes in a unified package to increase crop yield for approximately 50 years without considering the soil fertility status and crop demand. This emphasizes the importance of developing alternative methods to meet the nutrient needs of plants. In addition to the commonly used N and P fertilizers, mixed NPS contains S. However, the response of mung bean varieties to the amount of mixed NPS fertilizer in the Kindo Koysha district of the Wolaita district in Southern Ethiopia has not been studied. So, this study was done to investigate the effect of the NPS rate on yield and yield attributing traits of mung bean varieties.

## 2 Materials and methods

### 2.1 Description of the experimental site

The trial was carried out during the 2017/2018 harvest season at Kindo Koysha woreda (KKW), in the Wolaita Zone of Southern Ethiopia. KKW is geographically located at 6°79′7.06″ N latitude and 37°39′37.63″ E longitude 36 km southwest of the city of Wolaita Sodo to Jima. The altitude range of this area is 1,094 meter above sea level (m.a.s.l.). The study area is a semi-arid climate area with an average annual rainfall of around 400 mm. Rainfall has a bimodal pattern (*Meher* and *Belg*) of distribution, where the “*Belg*” planting season runs from February to June and the “Meher” season from July to October. The experimental site’s average maximum and minimum temperatures were 32 and 19.2°C, respectively, (unpublished report from the Kindo Koysha District Agricultural Office, 2018).

### 2.2 Treatments: design of experiments and field layout

Four NPS fertilizer rates (0, 50, 100, and 150 kg NPS ha^−1^ blended NPS (19% N, 38% P, and 7% S)) and four mung bean varieties were used in the experiments (Table 1). The experiment was laid out using a randomized complete block design (RCBD), and the treatments were repeated three times. The size of each plot was 2.1 m wide and 3 m long (6.3 m^2^ area), with a harvestable plot size of 3 m × 0.9 m (2.7 m^2^). The spacing between the plots and between blocks was 1 and 1.5 m, respectively. The row spacing and plant spacings were 30 and 10 cm, respectively. Each block has 7 rows, each row has 30 plants, and each block has 210 plants. Each row was sown with two seeds at a specified interval, with a depth of about 5 cm, to ensure full emergence. When planting, fertilizer was applied in rows by hand.

**Table 1:** Description of mung bean varieties used for the study

Varieties				
Characteristics	N26	Shewarobit	NVL-1	Chinese
Altitude (m.a.s.l.)	900–1,670	900–1,670	450–1,650	450–1,650
Rain fall (mm)	350–550	350–550	350–750	350–750
Fertilizer rate (kg ha^−1^)	P_2_O_5_ = 46	P_2_O_5_ = 46	P_2_O_5_ = 46	P_2_O_5_ = 46
	*N* = 18	*N* = 18	*N* = 18	*N* = 18
Maturity days	65–80	75–90	60–70	75–90
Yield on research (kg ha^−1^)	800–1,500	800–1,500	750–1,500	750–1,500
Yield on farmer (kg ha^−1^)	500–1,000	500–1,000	500–1,000	500–1,000
Year of release	2011	2011	2014	
Breeder	MARC	MARC	MARC	

### 2.3 Description of experimental materials

The blended NPS (19% N, 38% P, 7% S) used for this experiment was obtained from Woreda Agricultural Office. Mung bean varieties, namely, N26, Shewarobit, NVL-1, and Chinese, were used as planting materials. The description of the varieties used for this study is indicated in Table 1.

### 2.4 **Agronomic** p**ractice**

The experimental area was ploughed with a tractor-driven disc plough and cross-harrowed with the help of a disc harrow. Sowing was done in rows, with furrows opened by hand plough to a depth of 5 cm at a row spacing of 30 cm and plant to plant spacing of 10 cm. Fertilizers were applied as per treatments in each plot. Plant spacing was maintained by thinning out extra plants 15 days after sowing when all the plants emerged. Weed management is often done manually as needed. In addition, shallow cultivation was performed 25 days after emergence.

Harvesting was done at harvest maturity when the bottom of the mung bean pods started to dry [21]. The border area was first harvested and then removed from the experimental field. Later on, the net plot area was harvested. Produce from each net plot area was then bundled, tagged, and brought to the threshing floor for sun drying and threshing. After drying, the weight of the bundles (grain + straw) was recorded. Threshing was also done by beating the plants with the help of sticks, plot by plot. The weight of grain and straw was recorded treatment-wise and converted into kg ha^−1^.

### 2.5 Data collection and measurements

#### 2.5.1 Soil Sampling and Analysis

Prior to planting, 12 representative soil samples were randomly collected in a zigzag pattern from the entire experiment, with a depth of 0–30 cm. The sample was packed in bulk into a composite sample. The soil sample was air dried, crushed with a mortar, and passed through a 2 mm sieve.

The composite samples were analyzed for the soil texture, pH, organic carbon (OC), total N, cation exchange capacity (CEC), available K, P, S, B, and zinc in the Laboratory of Soil and Water Analysis in Debere Zeit, Ethiopia. A standard glass electrode pH meter was used to potentiometrically measure the soil pH value in a 1:2:5 soil water suspension [22]. The Walkley and Black method [23] was used to determine the organic matter content of the soil. The Kjeldahl [24] method was used to determine the total N in the soil. The Olsen extraction method was used to determine soil available P as described by Olsen and Dean [25]. Available S was analyzed by the turbidimetric method. The Bouyoucas hydrometer method [26] was used to determine soil texture. Electrical conductivity was measured with a standard glass electrode using an EC meter. The CEC of the soil was determined by the ammonium acetate method [27].

#### 2.5.2 Crop data

##### 2.5.2.1 Yield components and yield

The number of pods per plant was counted from the pods of the five plants sampled, and the average number was calculated and expressed as the number of pods per plant. Number of seeds per pod was recorded from ten pods of five sampled plants, and the average number was calculated and expressed as the number of seeds per pod. A representative sample of 1,000 grains of mung bean from each plot, sun-dried and moisture content adjusted to 10 %, was weighed in grams using a sensitive balance. Grain yield (kg ha^−1^) was estimated by weighing grains obtained after threshing, cleaning, and sun drying, and finally recorded in kg ha^−1^. Aboveground dry biomass yield (kg ha^−1^) was recorded by harvesting the three central rows in each subplot. The material was sun-dried up to a constant weight, weighed, and then converted into kg ha^−1^. Harvest index (HI) (%) was calculated as the ratio of economic yield (grain) and aboveground dry biomass yield. Its value was expressed as a percentage using the following formula:

\text{Harvest}\hspace{.25em}\text{index}\hspace{.25em}\text{(\%)}=\frac{\text{Grain}\hspace{.25em}\text{yield}\hspace{.25em}\text{(kg}\hspace{.25em}{\text{ha}}^{-1}\text{)}}{\text{Aboveground}\hspace{.25em}\text{dry}\hspace{.25em}\text{biomass}\hspace{.25em}\text{yield}\hspace{.25em}\text{(kg}\hspace{.25em}{\text{ha}}^{-1}\text{)}}\times 100.]

### 2.6 Economic analysis

The economic analysis was performed using the partial budget analysis procedure described by CIMMYT [28]. The cost of NPS and labor costs involved in applying NPS fertilizer and using it for this analysis are listed below. The current price of mung bean is calculated based on the average open market price of Kindo Koysha, which was 30 ETB per kg. The net income and other economic analyses are based on the formula developed by CIMMYT [28] and are given as follows:

The unadjusted grain yield (kg ha^−1^) was the average yield per treatment.

Adjusted grain yield (AGY) (kg ha^−1^) was the average yield adjusted downward by 10% to reflect the difference between the experimental and farmer yields. The total field profit (gross field benefits [GFB]) (ETB ha^−1^) was calculated by multiplying the field/farm price obtained by farmers when they sell their crops by the adjusted yield. GFB = AGY × The field/farm price of the crop.

Total variable cost (TVC) (ETB ha^−1^) was calculated by adding up the costs that vary, including the cost of NPS (14 ETB kg^−1^) fertilizers at the time of planting (August 1, 2018). The costs of other inputs and production practices, such as labor costs for land preparation, planting, weeding, chemical spraying, harvesting, and threshing, were considered the same for all treatments or plots.

Net benefit (NB) (ETB ha^−1^) was calculated by subtracting the TVCs from GFB for each treatment. GFB – TVC = NB

By dividing the change in NB by the change in TVC and multiplying by 100, the marginal rate of return (MRR) (%) was calculated.

### 2.7 Data analysis

Analysis of variance of the data collected was performed according to the general linear model procedure of SAS version 9.0 [29] appropriate to factorial experiments in RCBD, and interpretations were made following the procedure described by Gomez and Gomez [30]. Whenever a treatment effect was found to be significant, the least significant difference (LSD) test at the 5% significance level was used to compare the average.

## 3 Results

### 3.1 Physico-chemical characteristics of the experimental soil

Study site soil’s physical and chemical properties analysis results before planting, such as particle size distribution (texture), pH, OC, total N, available P, available K, available Zn and B, available S, and CEC are shown in Table 2. The particle size distribution of the experimental soil was found to be 26% sand, 38% silt, and 36% clay (Table 2). According to Rayan and Rashid [31], the soil at experimental fields was clay loam in textural class.

**Table 2:** Physico-chemical properties of the experimental site soil before planting 3

Soil properties	Results	Rating	Reference
Soil particle size	Sand (%) 26		
	Silt (%) 38		
	Clay (%) 36		
Textural class	Clay Loam		
Soil pH	6.23	Moderate	[32]
OC (%)	2.08	Moderate	[33]
Available N (kg ha^−1^)	0.13	Moderate	[34]
Available P (mg kg^−1^) (ppm)	9.48	Low	[34]
Available S (mg kg^−1^) (ppm)	37.76	Moderate	[34]
CEC (meq/100 g soil)	20.39		

The soil reaction of the experimental site was 6.23. This indicates suitability of the soil reaction in the experimental site for optimum mung bean growth and yield. Soil analysis before sowing indicated that the soil has medium level of total N, available S, organic matter, and low level of available P. The OC content (2.08 %) and total N (0.13 %) of the soil were medium. The level of available P content of the experimental soil was low (9.48 ppm) (Table 2). According to the rating of Landon [32] and Hazelton and Murphy [33], the N and P of the study site were poor requiring application of nitrogenous and phosphoric fertilizers. Overall, the fertility status of the study sites was low (Table 2).

### 3.2 Yield components and yield

#### 3.2.1 Number of pods per plant

**Figure 1:**
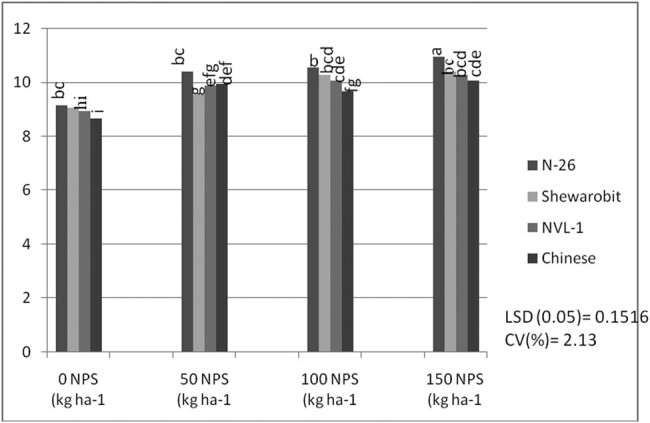
Interaction effects of varieties and NPS fertilizer rate on pod number per plant of mung bean at Kindo Koysha in 2018. LSD (0.05) = Least significant difference at 5% level; CV (%) = Coefficient of variation. Means in the table followed by the same letters are not significantly different at 5% level of significance.

Data analysis shows the interaction between NPS and varieties also significantly affected the number of pods plant^−1^. The average of the data shows that the variety N26 (15.46) produced the largest number of pods plant^−1^ at 150 kg ha^−1^, followed by the Chinese pod (14.96) plant at 150 kg ha^−1^, while the variety NVL1 provided the lowest number of pods per plant (9.83) with 0 kg  NPS ha^−1^ (Figure 1).

#### 3.2.2 Number of seeds per pod

**Figure 2:**
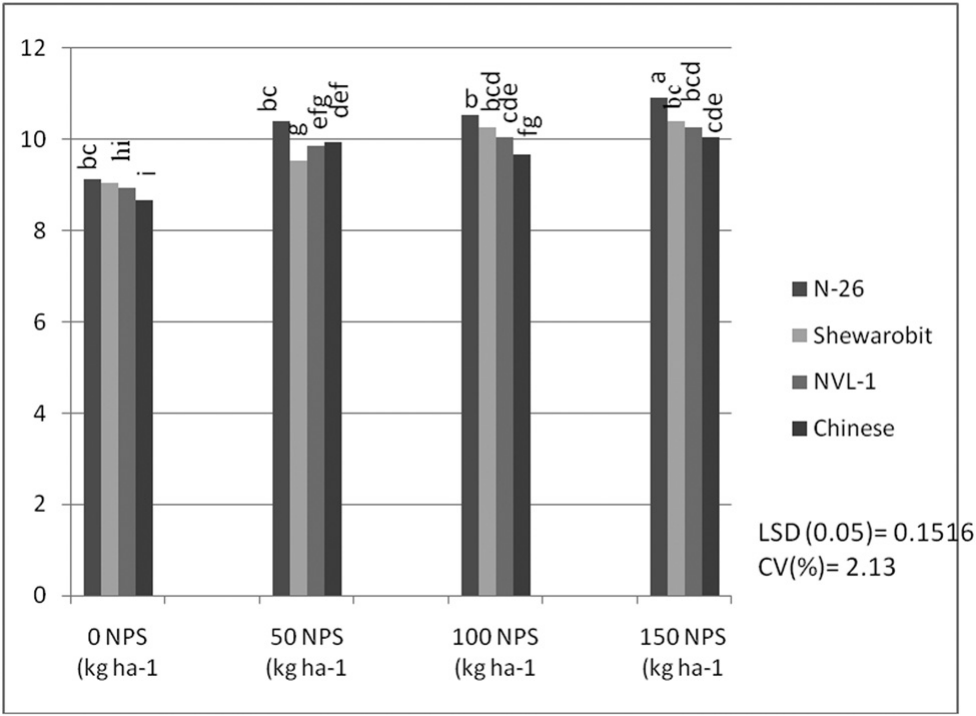
Interaction effects of varieties and NPS fertilizer rate on seed number per pod of mung bean at Kindo Koysha in 2018. LSD (0.05) = Least significant difference at 5% level; CV (%) = Coefficient of variation. Means in the table followed by the same letters are not significantly different at 5% level of significance.

The interaction between NPS level and mung bean variety had a significant (*P* < 0.05) effect on the number of seeds per pod. The highest number of seeds per pod (10.93) was recorded in the 150 kg NPS ha^−1^ level for variety N26, which was statistically the same at 100 kg ha^−1^ for the same variety (10.53), followed by all other combined treatments. The lowest number of seeds per pod (8.66) was recorded in the 0 kg ha^−1^ NPS level for Chinese variety (Figure 2). The change in the NPS level relative to the control (0 kg ha^−1^) indicated that the number of seeds per pod increased with the increase in the NPS level for the N26 variety.

#### 3.2.3 100-seed weight

The results revealed that thousand seed weight (TSW) was significantly affected by the rate of NPS and variety. The highest TSW (48.81 g) was recorded at a rate of 150 kg ha^−1^ NPS, which was statistically equivalent to 100 kg  ha^−1^ NPS (48.16 g) and 50 kg  ha^−1^ NPS (46.91 g), while the lowest TSW (43.57 g) was recorded for plots supplied with nil NPS fertilizer (Table 3). Further, the improved variety Chinese had the highest TSW (53.96 g), while the variety Shewarobit had the lowest TSW (40.19 g) (Table 3). This variance could be due to genotypic differences between the types.

**Table 3:** The main effect of NPS fertilizer rates and varieties on TSW of mung bean at Kindo Koysha in 2018

Treatment	TSW
**NPS rate (kg ha** ^**−1**^)	
0	43.57^b^
50	46.91^a^
100	48.16^a^
150	48.81^a^
LSD (0.05)	1.99
**Varieties**	
N26	50.55^b^
Shewarobit	40.19^d^
NVL-1	42.75^c^
Chinese	53.96^a^
LSD (0.05)	1.99
CV(%)	5.11

LSD (0.05) = Least significant difference at 5% level; CV (%) = Coefficient of variation. Means.

In the table followed by the same letters are not significantly different at 5% level.

#### 3.2.4 Grain yield

**Figure 3:**
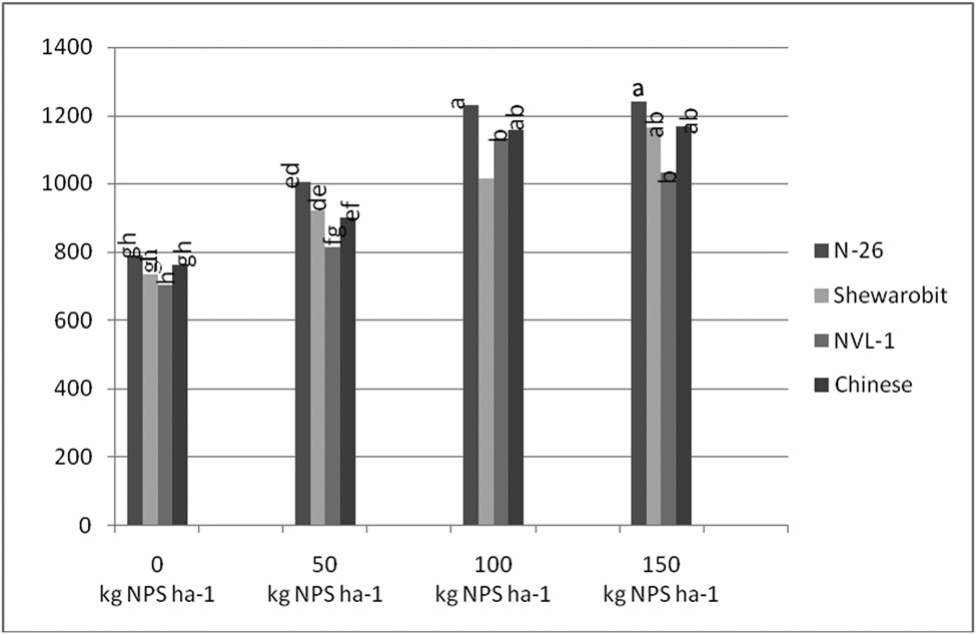
Interaction effects of varieties and NPS rates (kg ha^−1^) on grain yield of mung bean at Kindo Koysha in 2018. LSD (0.05) = Least significant difference at 5% level; CV (%) = Coefficient of variation. Means in the table followed by the same letters are not significantly different at 5% level of significance.

Grain yield was significantly (*P* < 0.05) affected by the interaction effect of the NPS level and the mung bean variety. Compared with the control, with the increase in NPS level, grain yield gradually increased. Variety N26 (1240.7 kg ha^−1^) recorded the highest grain yield at 150 kg  NPS ha^−1^, followed by the same variety N26 (1230.9 kg ha^−1^) at a NPS blending level of 100 kg ha^−1^, while the lowest grain yield (702.4 kg ha^−1^) was from the control for Chinese variety (Figure 3).

#### 3.2.5 Above ground biomass

**Figure 4:**
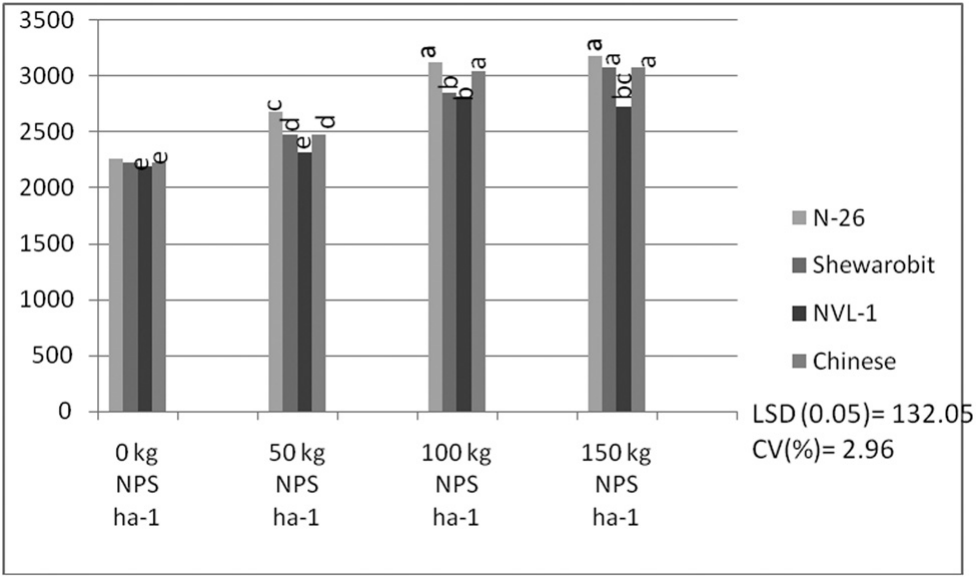
The interaction effect of varieties and NPS fertilizer rate on aboveground biomass (kg ha^−1)^ of mung bean at Kindo Koysha in 2018. LSD (0.05) = Least significant difference at 5% level; CV (%) = Coefficient of variation. Means in the table followed by the same letters are not significantly different at 5% level of significance.

Results revealed that the interaction between mung bean variety and NPS fertilizer rate had high significant impact (*P* ≤ 0.01) on the aboveground biomass yield. Variety N26 with 150 kg  ha^−1^ NPS produced significantly higher aboveground biomass yield (3177.40 kg ha^−1^), which was statistically equivalent to the same variety N26 (3121.50 kg ha^−1^) applied with 100 kg ha^−1^ NPS, while variety NVL1 applied with 0 kg  ha^−1^ NPS provided the lowest aboveground biomass yield per hectare (2193.4 ha^−1^), which is statistically equivalent to other varieties at the control level (Figure 4).

#### 3.2.6 HI

**Figure 5:**
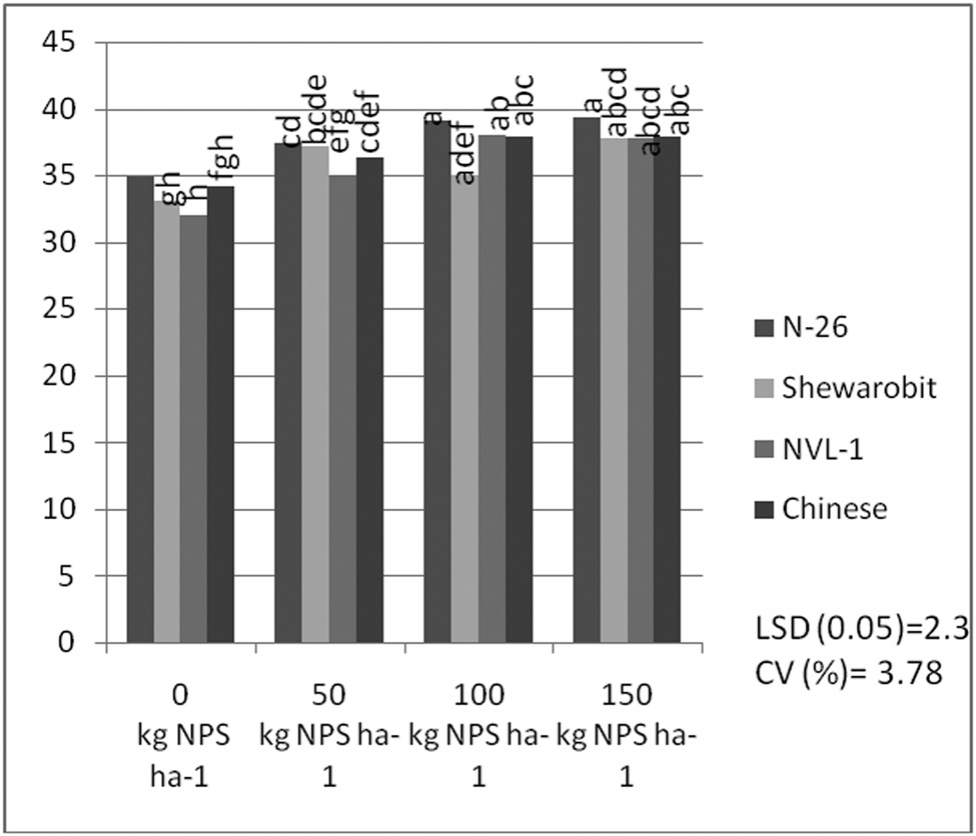
Interaction effects of varieties and NPS fertilizer rate on HI of mung bean at Kindo Koysha in 2018.

The results showed that interaction between variety and NPS rate had significant impact on the HI. Accordingly, the highest HI value (39.40%) came from the N26 variety at rate of 150  kg ha^−1^ NPS, while the lowest HI (32.02%) was for the NVL1 variety at the control plot (Figure 5).

#### 3.2.7 Partial budget analysis

Cost-benefit analysis results showed that the maximum NB (31,734.30 Birr ha^−1^) was obtained from variety N26 at an application rate of 100 kg  ha^−1^ NPS with MRR of 771.71% (Table 4). The feasibility of any method can be judged on the basis of additional performance generated by practice that exceeds established practice. Therefore, this is the ultimate deciding factor when choosing a profitable production technology. Furthermore, the use of any crop production technology by farmers to increase crop yield is completely dependent on their economy.

**Table 4:** Summary of partial budget analysis of the response of mung bean varieties to the application of NPS fertilizer at Kindo Koysha in 2018

Treatment combination	Unadjusted yield (kg ha^−1^)	Adjusted yield (kg ha^−1^)	Total revenue (Birr ha^−1^)	TVC (Birr ha^−1^)	Net return (Birr ha^−1^)	MRR (%)
N26 + 0	787.7	708.93	21267.90	0	21267.90	
N26 + 50	1004.9	904.41	27132.30	800	26332.30	633.05
N26 + 100	1230.9	1107.81	33234.30	1,500	31734.30	771.71
N26 + 150	1240.7	1116.63	33498.90	2,200	31298.90	D
Shewarobit + 0	734.6	661.14	19834.20	0	19834.20	D
Shewarobit + 50	921	828.9	24867.00	800	24067.00	529.1
Shewarobit + 100	1016.1	914.49	27434.70	1,500	25934.70	266.81
Shewarobit + 150	1164.2	1047.78	31433.40	2,200	29233.40	471.24
NVL-1 + 0	702.5	632.25	18967.50	0	18967.50	D
NVL-1 + 50	812.3	731.07	21932.10	800	21132.10	270.58
NVL-1 + 100	1130.9	1017.81	30534.30	1,500	29034.30	1,128.89
NVL-1 + 150	1167.9	1051.11	31533.30	2,200	29333.30	42.71
Chinese + 0	761.7	685.53	20565.90	0	20565.90	D
Chinese + 50	901.2	811.08	24332.40	800	23532.40	370.81
Chinese + 100	1130.9	1017.81	30534.30	1,500	29034.30	785.99
Chinese + 150	1167.9	1051.11	31533.30	2,200	29333.30	42.71

MRR (%) = Marginal rate of return; Fertilizer application cost = 100 Birr ha^−1^; NPS cost = 14.00 Birr kg^−1^; Mung bean grain local selling price = 30 Birr kg^−1^; TVC = Total variable cost; D = Dominated treatment.

## 4 Discussion

### 4.1 Yield components and yield

**Figure 6:**
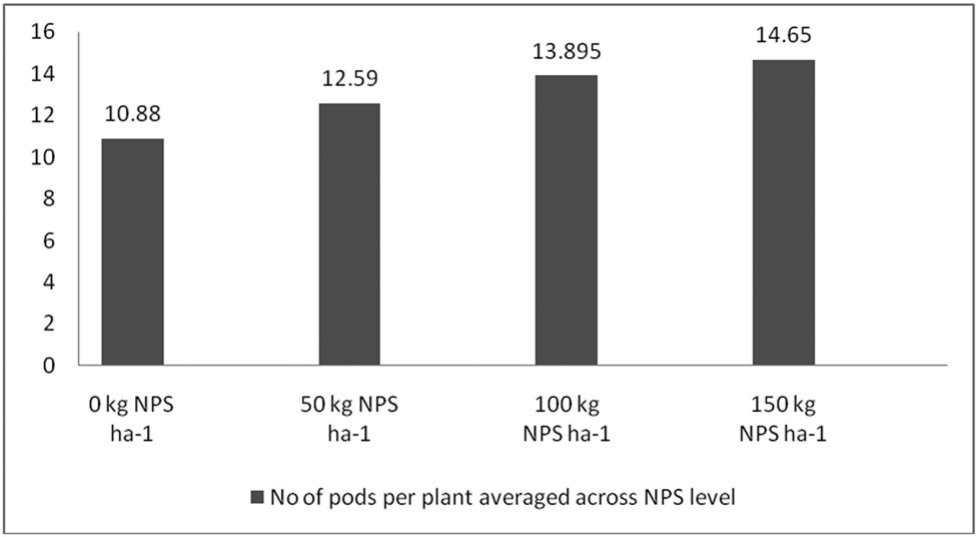
Number of pods per plant averaged across NPS level.

**Figure 7:**
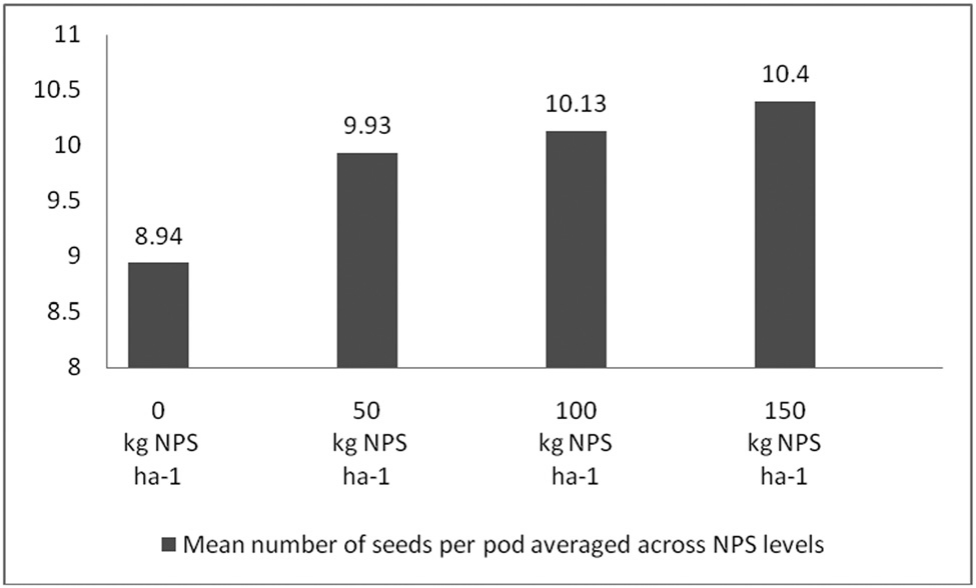
The mean number of seeds per pod averaged across NPS level.

**Figure 8:**
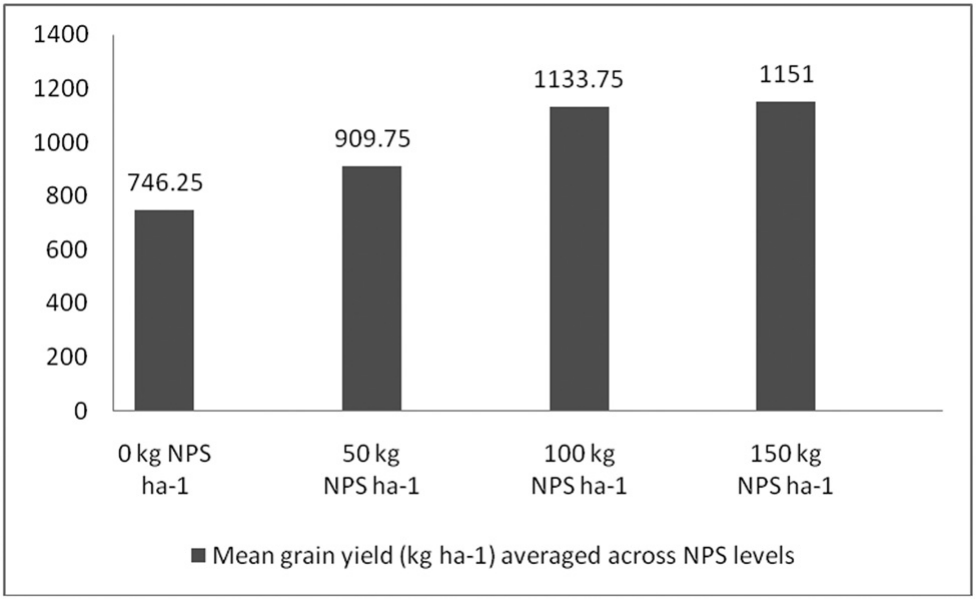
Mean grain yield (kg ha^−1^) averaged across NPS level.

**Figure 9:**
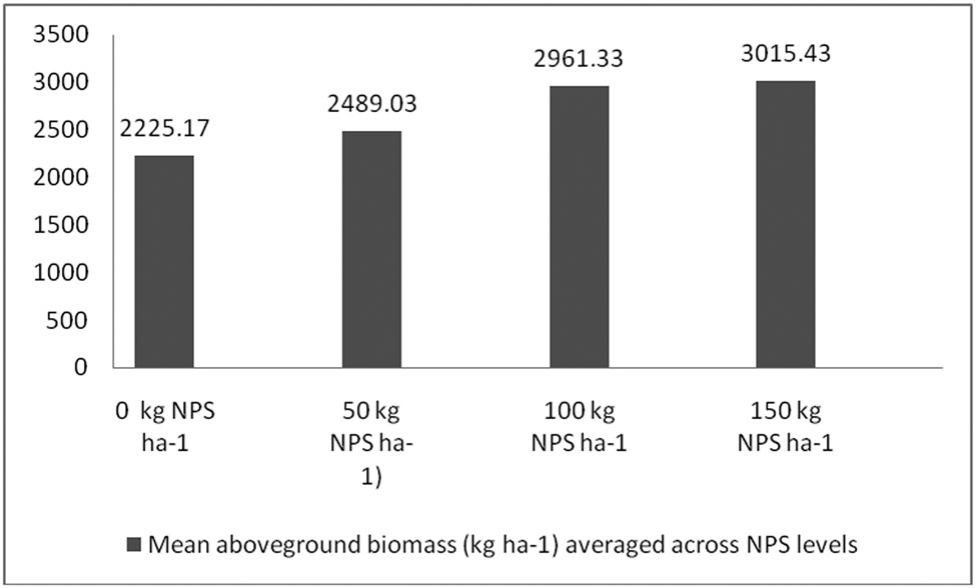
Mean aboveground dry biomass averaged across NPS level.

Correcting soil nutrient deficiencies is among the strategies to ensure the higher productivity of legumes in Ethiopia. It is also well documented that N, P, and S are indispensable plant nutrients for plant growth and productivity [35,36,37], and their deficiency can severely affect plant productivity. Nevertheless, the pre-plant analysis of the soils of the study area indicated inadequate availability of N, P, and S nutrients (Table 2). In this regard, applying the optimum rate of the newly introduced blended NPS fertilizer was considered an immediate solution to correct the soil nutrient deficiencies in the country in general and in the study area in particular. To this end, the present study also showed that, averaged across varieties, the maximum number of pods per plant, number of seeds per pod, 100-seed weight, aboveground biomass weight, and grain yield were produced at the highest blended NPS (150 kg ha^−1^) rate (Table 3, Figures 6–9). Regardless of the varietal difference, NPS application improved the productivity of mung bean in the study area.

The results also revealed that mung bean varieties produced a significantly varying number of pods and seeds per plant across different levels of NPS fertilizer (Figures 1 and 2). This indicates a difference in nutrient requirements among the varieties. Previous studies also indicated a significant interactive effect of blended NPS fertilizer level and varieties on the number of pods per plant [38,39] and seeds per pod [40]. Number of pods per plant showed a 57% increase in pods per plant at 150 kg ha^−1^ NPS over nil rates of NPS for variety NVL-1, which is a low yielding variety. However, N26 attained the optimum number of pods per plant at 100 kg ha^−1^ NPS. There is an increasing trend in the number of pods per plant as the NPS level increases from zero to 150 kg ha^−1^. This differential response among the varieties might be related to their genotypic differences.

The results also revealed that mung bean varieties also produced significantly different(TSW) (Table 3). A 34% increase in 100-seed weight was recorded for the variety producing the higher 100-seed weight than the variety producing the lower 100-seed weight. This variation in TSW could be due to genotypic differences between the varieties. Supporting the present finding, Oljirra and Temesgen [41] also reported a significant difference among soybean varieties in 100-seed weight. Further, the findings of this study also indicated that the application of NPS significantly affected the TSW of mung bean. Corroborating the results, different scholars reported that application of N and P fertilizers can significantly influence the seed weight of different legumes, including mung bean [42,43,44]. For instance, Yin et al. [42] reported that N and P fertilizers had extremely significant (*P <* 0.01) effects on the 100-seed weight of mung bean.

Mung bean varieties also produced significantly different aboveground biomass yield across NPS level (Figure 4). The higher aboveground biomass yield (3177.40 kg ha^−1^) was produced by variety N26 at 150 kg ha^−1^ NPS. Averaged across mung bean varieties, the highest aboveground biomass was produced by N26. Nearly 45% increase in aboveground biomass over variety NVL-1 at nil NPS rate was recorded for variety N26 at 150 kg ha^−1^ NPS. This result is consistent with that of Girma [45], who found that increasing NP fertilizer rates from 0 to 27 kg ha^−1^ N, 0 to 69 kg ha^−1^ P_2_O_5_ (150 kg DAP) enhanced common bean biological yield. In line with this finding, Shanka et al. [46] found that the three-factor interaction impact of site X cultivar X P was significant for aboveground dry biomass production and different dry matters at different P levels on common bean cultivars at varied P levels. The increased biomass yield of cultivars could be attributed to genotypic variations in cultivars across blended NPS rates, or to the fact that increased N availability increased plant height, number of pods per plant, and overall vegetative growth of the plants, all of which contributed to higher aboveground dry biomass yield.

Grain yield is a function of yield attributing traits such as number of pods per plant and seeds per pod, and 100-seed weight. The results indicated that nearly 77% higher grain yield over the low yielding variety NVL-1 at nil rates of NPS was produced by N26 variety at the highest rates of NPS (Figure 3). However, applying NPS in excess of 100 kg ha^−1^ to variety Shewarobit did not result in a significant yield enhancement. In other words, using more than 100 kg of NPS to increase production of the Shewarobit mung bean variety at Kindo Koysha is not recommended. Further, averaged across mung bean varieties, N26 produced the highest grain yield, while the lowest was produced by Chinese variety. Averaged across the NPS levels also the highest gained yield was produced at 150 kg  ha^−1^ NPS, while the lowest was produced at nil application rates. The findings are consistent with Nebret [47], who found that greater rates of N above 46 kg ha^−1^ have negative impacts on grain production of common bean. This could be due to a tendency to increase vegetative growth, which could have resulted in self-shading, lowering overall output.

Significantly different HI was recorded for mung bean varieties supplied with different levels of NPS fertilizer (Figure 5). The reason for the higher value of HI may be due to the greater physiological potential of the crop in partitioning efficiency of dry matter into economic yield and increase in seed yield following application of NPS, accompanied by increased HI. Further, this study is consistent with the study by Kawte et al. [20], who recorded higher HI, at higher NPS rate for mung bean varieties.

In general, averaged across the NPS level, the highest number of pods per plant, number of seeds per pod, 100-seed weight aboveground biomass, and grain yield were recorded at the highest NPS level (150 kg ha^−1^). The production of a higher number of pods per plant and seeds per pod, 100-seed weight, and grain yield at the highest NPS rates (150 kg ha^−1^) could be due to enhanced photosynthetic activity, leaf area development, and dry matter production [36,46]. As a result, this in turn could have enhanced better photo-assimilate production and translocation into reproductive parts, enhancing the formation of pods, seed setting, seed weight, and economic or grain yield [20,47]. For instance, Seid et al. [48] noted an increase in the number of pods per plant of chickpea as a result of increased leaf area, with more N being associated with more reproductive nodes.

There are immense research reports regarding the important roles of N, P, and S in photosynthetic processes [49,36,37]. In their study, Jiaying et al. [36] noted higher net photosynthetic rate, ATP content, as well as NADH dehydrogenase, cytochrome oxidase, and ATPase activities, for plants supplied with adequate N and P. Phosphorus is involved in the energy transfer process in photosynthesis and the synthesis of chlorophyll [50,51], whereas S plays an important role in the biosynthesis of chlorophyll and proteins and enzyme activation. This indicates that the improvement in yield and yield related traits could be attributed to the improvement in photosynthetic processes following application of blended NPS fertilizer.

Besides, P is thought to be vital for seed formation and development. It is also a component of phytin, which is a major storage form of P in seeds [51]. Hence, the increase in the 100-seed weight with the levels of NPS might be attributed to a direct and positive contribution of P to the formation and development of the seed.

From an economical point of view, producers or farmers should maximize their profit from their produce. In other words, they should get an economically acceptable return for their investment. The minimum acceptable MRR should be 100%, according to CIMMYT [28]. Based on this, the economic analysis of this study also indicated that the higher net incomes with acceptable marginal returns were obtained using the N26 mung bean variety. The most attractive NPS fertilizer application rate is 100 kg ha^−1^. In agreement to this study, Shumi et al. [52] reported that the economic analysis showed that the application of 150 kg ha^−1^ NPS can obtain the highest NB (34,167.56 Birr ha^−1^), while application of nil NPS resulted in the lowest NB (19,228.69 Birr ha^−1^). Therefore, the amount of fertilization mentioned above is recommended as the best economic return.

## 5 Conclusion

The results of this study evidenced that mung bean varieties can vary in terms of their blended NPS fertilizer requirement. Hence, the NPS fertilizer management should be designed accordingly. Further, the overall improvement in, yield determining traits such as number of pods per plant, seeds per pod and 100-seed weight resulted in higher grain yield by the best performing variety N26 at the highest blended NPS fertilizer rate (150 kg ha^−1^). Hence, based on the present findings it can be concluded that application of optimum rate of blended NPS fertilizer along with best performing mung bean varieties can boast of high grain yield of mung bean and income to the farmers in the study area. However, based on economic analysis, applying NPS at 100 kg ha^−1^ resulted in higher NB. Accordingly, growing variety N26 at application rate of 100 kg ha^−1^ was found to be economically optimum rate for mung bean production in the study area and other similar agroecologies elsewhere in the country or around the globe.
